# hiPSC-based models to decipher the contribution of human astrocytes to Alzheimer’s disease and potential therapeutics

**DOI:** 10.1186/s13024-023-00612-9

**Published:** 2023-03-25

**Authors:** Julia TCW, Amaia M. Arranz

**Affiliations:** 1 Department of Pharmacology, Physiology & Biophysics, Boston University, Chobanian & Avedisian School of Medicine, Boston, USA; 2 Graduate Program of Neuroscience, Boston University, Boston, USA; 3 Graduate Program of Bioinformatics, Boston University, Boston, USA; 4 Achucarro Basque Center for Neuroscience, Bilbao, Spain; 5 Ikerbasque Basque Foundation for Science, Bilbao, Spain

Astrocytes constitute a large part of the brain cell mass and play essential functions in the central nervous system. They provide trophic and metabolic support to neurons, regulate synapse formation, neurotransmission, calcium homeostasis, and control immune response and blood flow. In Alzheimer’s disease (AD), astrocytes undergo profound molecular, morphological and functional alterations that arise at early stages and exacerbate as disease evolves, indicating that astrocytes transition from homeostatic to dysfunctional disease-associated states and pointing to these cells as critical contributors to AD progression. Genome-wide association studies (GWAS) support this idea as many genes associated with increasing the risk of developing late-onset AD (LOAD) are expressed in glial cells: astrocytes, microglia and oligodendrocytes [1]. Among those, major AD risk genes such as *APOE*, *CLU* and *FERMT2* are predominantly expressed in astrocytes [1]. While our current knowledge of astrocyte contribution to AD is mainly coming from studies in mouse models, critical species-specific differences highlight the importance of studying astrocyte (dys)function in human-based systems. The ability to generate human induced pluripotent stem cells (hiPSCs) from patients and differentiate them into astrocytes is providing exciting opportunities to explore their functions in AD. We summarize recent studies in 2D and 3D cultures that provide essential clues on the impact of human astrocytes on AD and propose potential astrocyte-targeting therapeutics.

**Fig. 1:**
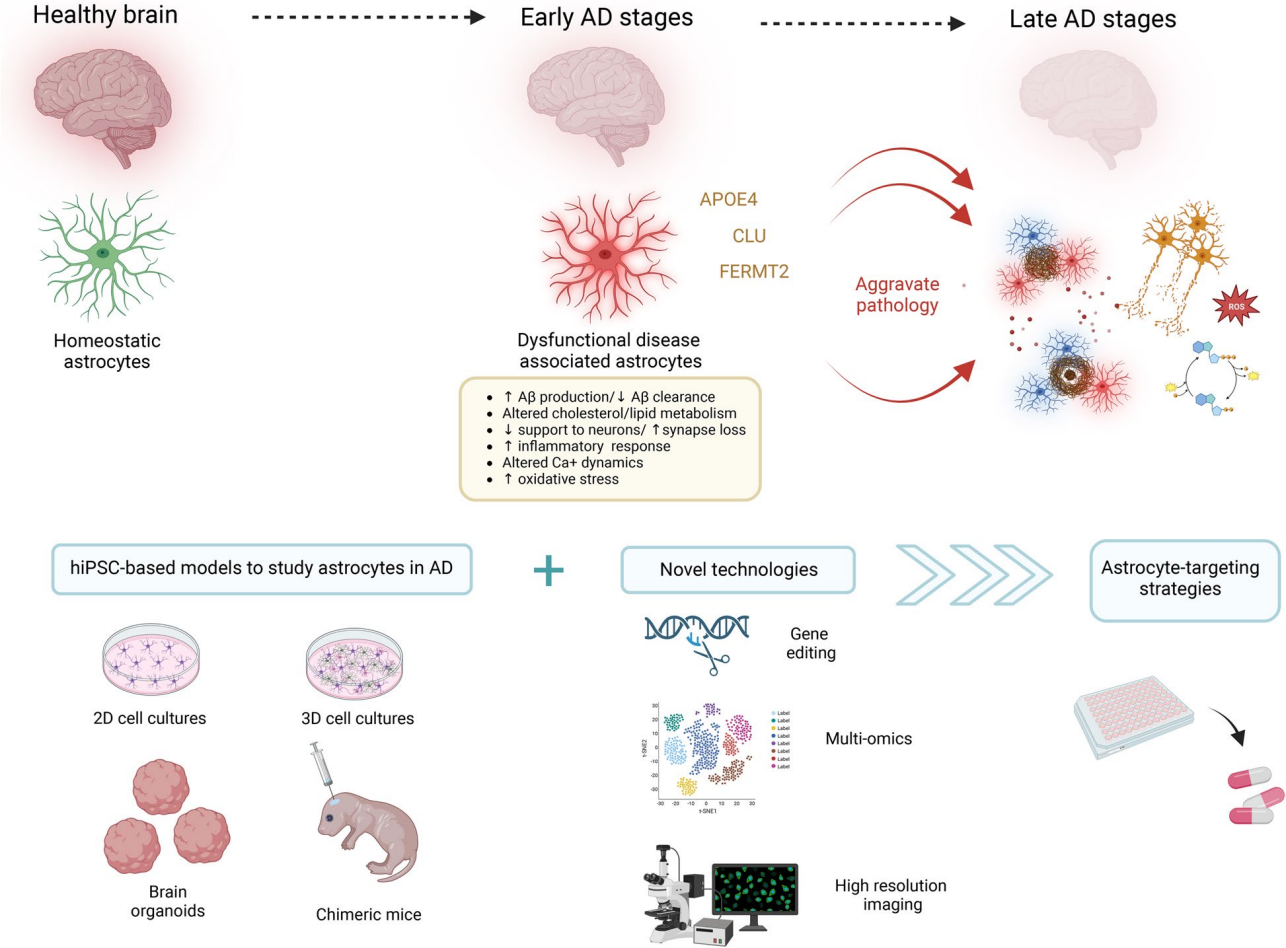
Human astrocytes get dysfunctional at early stages of AD and exacerbate the pathology affecting major AD processes. Combination of hiPSC-based in vitro and in vivo models with gene editing, multi-omics technologies and high-resolution imaging will allow in depth analyses of human astrocytes in AD and enable the development of astrocyte targeting therapeutics. Created with BioRender.com

Earlier studies in 2D models revealed that healthy hiPSC-derived astrocytes play important roles in APP processing, secrete β-amyloid (Aβ) [2], and therefore have the potential to contribute to Aβ accumulation in AD brains. Astrocytes derived from early-onset AD patients carrying the *PSEN1ΔE9* mutation display AD hallmarks including increased Aβ and reactive oxygen species (ROS) production, altered inflammatory responses and dysregulated calcium homeostasis compared to isogenic control astrocytes [3]. When co-cultured with *PSEN1* mutant astrocytes in a 3D system, isogenic control neurons display alterations in calcium signaling [3], which highlights a major impact of human astrocytes on human neuron physiology and functionality. Interestingly, activation of NF-E2-related factor 2 (NRF2), key regulator of antioxidant defense pathways, reduces Aβ secretion and modulates cytokine release and oxidative stress in *PSEN1* mutant astrocytes [4], suggesting that targeting NRF2 in AD astrocytes could be a potential therapeutic strategy.

Astrocytes are the major cell type expressing and producing Apolipoprotein E (APOE) in the brain, although microglia, particularly in an activated state, also produce APOE [1, 5]. APOE facilitates the transport of lipids and cholesterol to neurons, microglia and oligodendrocytes, and binds to Aβ plaques [6]. The *APOE* gene has three polymorphic alleles (ε2, ε3 and ε4), and *APOE4* confers the greatest risk for LOAD at an earlier age of disease onset in a gene dose-dependent manner. Recent studies have started to elucidate how *APOE* affects human astrocyte functions in the context of AD. hiPSC-derived astrocytes carrying the *APOE4* variant produce and secrete less APOE protein compared to *APOE3* astrocytes, are less efficient at clearing extracellular Aβ, and show impaired lipid/cholesterol metabolism [5, 6]. Astrocytes produce the majority of the brain cholesterol, and increased cholesterol biosynthesis has been found in *APOE4* astrocytes and microglia [6] as well as in organoids from individuals carrying Tau mutations [7]. These data suggest that perturbed cholesterol metabolism is a common pathway and early event in the etiology of AD. Besides, *APOE4* astrocytes show increased inflammatory responses compared to *APOE3* astrocytes [6]. A recent study describes an exacerbated proinflammatory state on *APOE4* astrocytes associated with Transgelin 3 (TAGLN3) downregulation and NF-kB activation [8]. Interestingly, this state can be pharmacologically reverted by TAGLN3 supplementation, highlighting TAGLN3 as a target to modulate inflammation in *APOE4* astrocytes. Importantly, cell-autonomous imbalances in astrocytes can also affect astrocyte-neuron communication. When co-cultured with neurons, *APOE4* astrocytes provide less support than *APOE3* astrocytes for neuronal survival and synaptogenesis, while exacerbating neuroinflammation [6, 9]. Moreover, in organoids containing either *APOE3* or *APOE4* neurons and astrocytes there is an increased *APOE4*-dependent synapse loss, neurodegeneration, and Tau pathology [5, 10].

Astrocyte-microglia crosstalk also has an impact on AD-associated inflammatory processes. In tri-culture systems with healthy hiPSC-derived astrocytes, neurons and microglia, the complement protein C3, which is elevated in brains of AD patients and involved in neurodegeneration, increases under inflammatory conditions due to astrocyte-microglia reciprocal signaling that induces them to overproduce C3 [11]. Astrocytic production of C3 induced by microglia, as well as microglial production of C3 re-induced by astrocytes are further enhanced in AD tri-cultures derived from hiPSCs harboring the *APP*^*SWE*^ mutation [11]. Another 3D tri-culture AD model that develops Aβ pathology and Tau accumulation shows that astrocyte-secreted interleukin-3 (IL-3) reprograms microglia at molecular, morphological and functional levels [12]. Reprogrammed microglia acquire an acute immune response, increased motility and enhanced capacity to cluster and clear Aβ and Tau aggregates restricting AD pathology [12]. Astrocytes secrete other inflammatory factors and molecules potentially involved in their crosstalk with microglia, neurons and other brain cells. hiPSC-based in vitro systems are well-suited for modeling the inflammatory axis among these cells, dissecting the role of astrocytes in complex AD-associated cellular environments and assessing their impact on neurodegeneration in AD. Moreover, these models constitute a powerful approach for identifying key compounds and main molecular players enabling the development of astrocyte targeting therapeutic strategies.

In summary, human astrocytes become dysfunctional at early stages of AD and aggravate pathology affecting major processes including Aβ production and clearance, cholesterol and lipid metabolism, oxidative stress and calcium dynamics. Moreover, in AD, human astrocytes lose their homeostatic functions providing less support to synapses and neurons, and closely interact with microglia, being key mediators of neuroinflammation (Fig. 1). Novel therapeutic strategies targeting astrocytes should be aimed at improving their neurotrophic and neuroprotective properties, modulating their immune responses, and reversing their cholesterol and lipid metabolic alterations, oxidative stress and calcium dysfunction.

Although 2D and 3D in vitro models with hiPSC-derived astrocytes are advancing our understanding of the cellular and molecular processes involved in AD, they lack important tissue components, have limited maturation and are not able to recapitulate the vast complexity of the human brain. These limitations can be partially overcome by next-generation approaches based on analyzing human astrocytes in vivo, transplanted into the brain of chimeric AD model mice [13]. Chimeras offer a unique opportunity to analyze human astrocyte (dys)function in the live brain and to understand how it contributes to AD progression. Combination of in vitro and in vivo models with CRISPR/Cas9 editing, which allows targeting specific AD risk genes in human astrocytes, multi-omics technologies and high-resolution imaging (Fig. 1) will be critical for further exploring the role of human astrocytes in AD, and it will open new avenues enabling the development of astrocyte-specific targeted therapeutics to prevent, slow down and even cure AD.

## Data Availability

Not applicable.

## Abbreviations

AD: Alzheimer’ s disease
GWAS: Genome-wide association studies
LOAD: Late-onset AD
hiPSCs: Human induced pluripotent stem cells
Aβ: β-Amyloid
ROS: Reactive oxygen species
*APOE*: *APOE* gene
APOE: Apolipoprotein E protein
TAGLN3: Transgelin 3
IL-3: Interleukin-3
